# Communication about incurable illness and remaining life between spouses and patients with incurable illness receiving specialized home care: effects of a family caregiver-targeted web-based psycho-educational intervention

**DOI:** 10.1186/s12904-024-01614-0

**Published:** 2024-12-16

**Authors:** Sandra Doveson, Louise Häger Tibell, Kristofer Årestedt, Maja Holm, Ulrika Kreicbergs, Anette Alvariza, Viktoria Wallin

**Affiliations:** 1 Department of Nursing Science, Sophiahemmet University, Box 5605, Stockholm, 114 86 Sweden; 2 The Department of Health Care Science, Marie Cederschiöld University, Stockholm, Sweden; 3 Tema Cancer, BES: Breast-Endocrine Tumours and Sarcoma, Karolinska University Hospital, Stockholm, Sweden; 4 Faculty of Health and Life Sciences, Linnaeus University, Kalmar, Sweden; 5 Louis Dundas Centre, Great Ormond Street Institute of Child Health, University College London, London, UK; 6 Department of Women’s and Children’s Health, Karolinska Institutet, Stockholm, Sweden; 7 Research and Development-Unit/Palliative Care, Stockholms Sjukhem, Stockholm, Sweden; 8 Department of Research, Region Kalmar County, Kalmar, Sweden

**Keywords:** Palliative care, Family caregivers, Web-based support, EHealth/digital support, Spouses, End of life, Communication

## Abstract

**Background:**

Web-based interventions targeted at family caregivers has become a quickly expanding research field, none the least since a growing number of patients with incurable illness are being cared for at home. Spouses, who are also family caregivers, constitute an especially vulnerable group in need of support when they are cohabitating with the ill patient and research shows that communication regarding the illness is important, yet challenging. This study therefore explored effects of a family caregiver-targeted web-based psycho-educational intervention on communication about incurable illness and remaining life between spouses and patients receiving specialized home care.

**Methods:**

The study had a pre-post-design. An intervention containing videos and texts about family caregiving was developed and made accessible via a website. Thirty-nine spouses (67% women, median age: 61) were recruited from specialised home care services. At baseline, and after 4 weeks of access to the website, spouses completed a questionnaire about communication with the patient regarding incurable illness and remaining life. Data was analyzed using the Wilcoxon signed-rank test.

**Results:**

No significant changes were found between baseline and follow-up. Most spouses did, however, report having talked with the patient about the illness being incurable (64%) and how the illness affected the patient physically (64%) and psychologically (77%) during the past month already at baseline. Regarding communication about the remaining life and how to manage once the patient had passed away, 46–59% instead reported not having had these conversations with the patient ever.

**Conclusions:**

A majority of the spouses had talked about aspects of the illness and its consequences already at baseline, indicating that these matters are important to spousal caregivers of patients with incurable illness. However, a sizeable portion had not ever talked to the patient about how to manage once the patient had passed away, suggesting there are barriers to such conversations that need to be further explored. Future research on web-based psychoeducational interventions targeted at family caregivers need to address barriers and the diverse support needs regarding communication, especially about the remaining life, among spouses of patients with incurable illness.

**Trial registration:**

The study was first registered on clinicaltrials.gov(NCT03676283) on 2018.09.12.

**Supplementary Information:**

The online version contains supplementary material available at 10.1186/s12904-024-01614-0.

## Introduction

Web-based interventions targeted at family caregivers is an expanding field, none the least since an increasing number of patients with serious illness are being cared for at home. The web-based interventions commonly consist of either educational components alone, or educational components alongside psychosocial support of some sort [1]. As opposed to face-to-face interventions, that have been proven beneficial to family caregivers [2], web-based support interventions could be especially suitable for family caregivers of patients with incurable illnesses [3]. There are several web-based interventions targeted at family caregivers of patients with incurable illnesses such as cancer [4] or dementia [5, 6]. The trajectory of an incurable illness, increasing palliative care needs and the approaching end of life and death inevitably impact everyday life for patients at the end of life and their families [7, 8]. Family caregivers of a person with an incurable illness often take on care responsibilities, and the scope of such responsibilities commonly increases as the illness progresses.

Caring for an ill family member has been described as both rewarding [9–11] and burdensome [12] and a recent study concluded that both family caregivers and the patients experience uncertainty and feel overwhelmed in the challenging everyday life when living close to, and with, incurable illness [13]. Spousal caregiving is considered the most demanding type of caregiving, primarily because it involves living together and often lasts for extended periods. Spousal caregivers typically experience higher levels of caregiver-related distress compared to caregivers who are adult children [14]. Further, for spouses, higher unmet support needs are associated with poorer quality of life [15]. When living with someone incurably ill, lack of support and confidence in caregiving are recurrently reported by family caregivers [9, 16]. The challenges associated with being a family caregiver can lead to negative consequences, such as ill-health [17]. Hence, many web-based interventions are aimed at improving the family caregivers’ experiences of e.g. caregiver burden, self-efficacy [1, 18] self-esteem, life satisfaction, caregiver strain, social support [1], emotional distress, quality of life [4] and caregiver knowledge [18]. Communication when a close one is suffering from an incurable illness has been described as challenging with several barriers. These include a wish to protect both oneself and the patient from malaise or discomfort, or avoidance of the topic of death due to fear, feeling unready or believing it could even hasten the patient’s death [19]. At the same time, an open communication regarding illness and death prior to the patient’s death has been shown to reduce the risk of bereavement distress in family caregivers of patients with incurable illness [20], which underlines the need for interventions to support communication.

Family caregivers face, and are expected to manage, not only the ill patient’s current situation but also their uncertain future and impending death and its aftermath [21]. In response, interventions are being designed to increase family caregivers’ preparedness for handling challenges associated with caring for, and losing, a close one [22]. There is, to our knowledge, however no research that explores the effects of web-based interventions on communication between incurably ill patients and spouses specifically. Hence, the aim of this study was to explore effects of a family caregiver-targeted web-based psycho-educational intervention on communication about incurable illness and remaining life between spouses and patients receiving specialized home care.

## Methods

### Design and setting

This study was designed to evaluate a family caregiver-targeted web-based psycho-educational intervention for family caregivers. It had a pre-post design, using questionnaire data. Five specialized home care services in an urban area of a large Swedish city constituted the base for participant recruitment. The services are staffed by multi-disciplinary teams, including registered and assistant nurses, physicians, physiotherapists, occupational therapists, dieticians, and social workers. They provide 24-h-a-day medical care for patients with palliative care needs. The care is provided individually based on each patient’s needs and can vary from home visits once a week, up to home visits several times a day. The Regional Ethical Review Board in Stockholm approved the study prior to its commencement (no. 2018/1893–31; 2019–02242; 2021–00235). The study is registered on clinicaltrials.gov (NCT03676283).

### The family caregiver-targeted web-based psycho-educational intervention

**Fig. 1:**
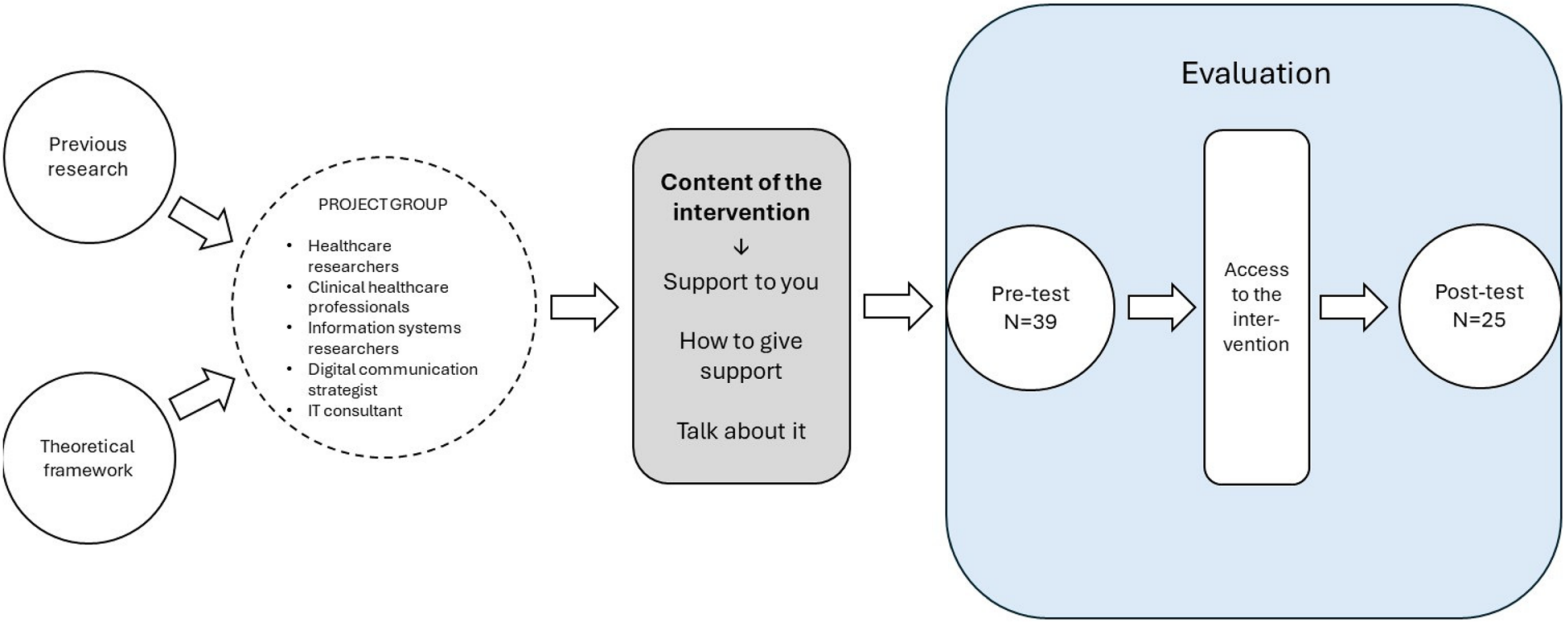
Development and evaluation of the intervention

The family caregiver-targeted web-based psycho-educational intervention is presented at the website “narstaende.se” [23] (“narstaende” translates approximately to “family caregiver” in English). The intervention is evidence-based and built on previous research [11, 24–28]. As theoretical foundation for the intervention, the framework of family caregivers’ principal needs “knowing, being and doing” by Andershed and Ternestedt [29] served as a guidance. The website was designed and developed in collaboration with healthcare researchers, clinical healthcare professionals, information systems researchers, digital communication strategists, and IT consultants, and is accessible via smartphone, computer, or tablet (Fig. 1).

**Fig. 2:**
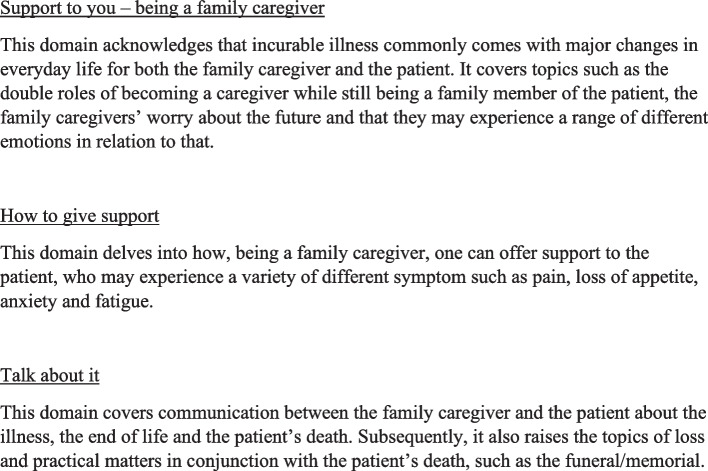
Examples of content within the three domains at the website “narstaende.se”

The intervention comprises short videos, informative texts, links to further information and an unmoderated, participant-driven chat forum. Altogether, there are 23 videos, ranging between 2.5 and 8.5 min (Tibell et al., 2022). The website content is structured into three main domains: “Support to you – being a family caregiver,” “how to give support,” and “talk about it,” covering topics such as practical and medical issues, communication, and considerations for the future (Fig. 2).

### Participants and procedure

**Fig. 3:**
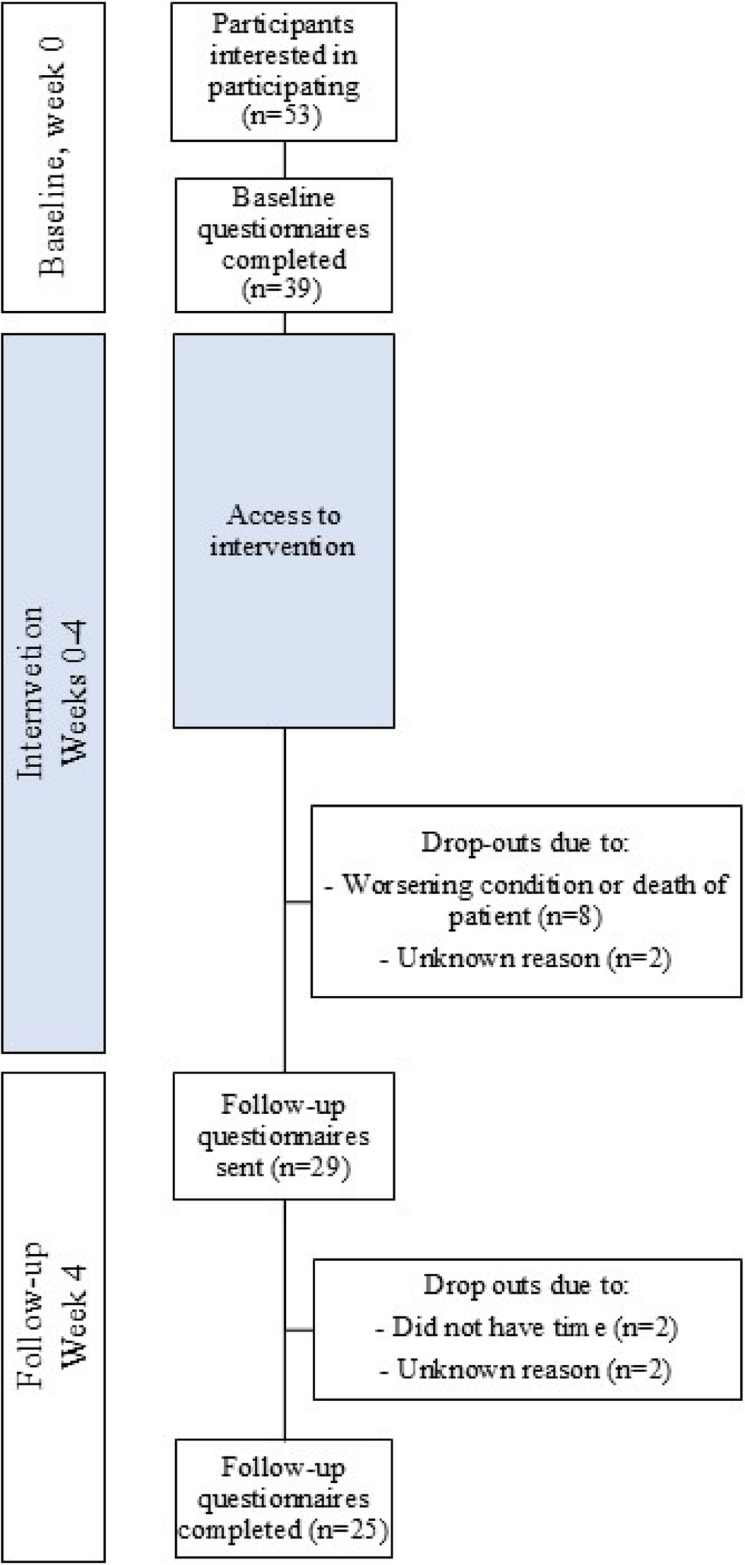
Study procedure and timeline

Swedish-speaking cohabitating spouses of patients with incurable illness and palliative care needs (both legal adults, ≥ 18 years of age), cared for in specialized home care, were included in the study. Spouse was defined as a wife, husband or partner cohabitating in a shared household with the patient. Eligible spouses were identified via patient medical records by the second author. Information letters for both the patient and spouse were sent via post to the patient, asking for permission to contact the spouse about participation in the study. The letter was shortly followed by a text message to set up a suitable time for a phone call. The spouse was then contacted via telephone by the second author, who provided oral information about the study. If the spouse consented verbally to participate, instructions for registration were given orally or if wished by e-mail. Upon registration at “narstaende.se,” the spouse provided written consent to participate, whereafter the baseline questionnaire was obtained at the website and needed to be answered prior to gaining access to the intervention at the website. After 4 weeks, a follow-up questionnaire was distributed via e-mail (Fig. 3).

### The questionnaire

The study questionnaire included study-specific questions about demographic data for spouses and patients, the spouses’ health and quality of life (QoL), and conversations with the patient. The single item questions have been developed based on experiences from psychologists, nurses, and physicians, researchers, and bereaved family members. They have previously been validated, using face-to-face methodology, and used in Swedish studies on bereaved family members [24, 30–32]. The demographic questions used in the present study included: age, gender, country of birth, educational level, employment status, duration of the relationship with the patient, and time since the patient´s diagnosis. Two questions covered the spouses’ physical and psychological health and one question their overall QoL. These three questions were responded on a seven-point scale, ranging from 0 “worst possible” to 6 “best possible”. To explore the study aim, six questions concerning communication with the patient about their own thoughts and feelings, the incurable illness, aspects of the remaining life and how the illness affects the patient were also used. These are presented in Table S1 (Supplementary material).

### Data analysis

Descriptive statistics was used to present demographic data and study variables. Non-ordered categorical data was presented with frequencies and proportions, ordered categorical data with frequencies or medians and quartiles, and continuous data with mean and standard deviations. To explore effects of the intervention, the Wilcoxon signed-rank test was used to compare the pre- and post-assessments, i.e., the six questions about the spouses’ communication with the patient. The non-parametric Wilcoxon signed-rank test was chosen due to ordinal-level data in these variables. Effect sizes were calculated using Cohen’s *r* (*r* = Z/√n) [33]. Statistical significance was set at *p* < 0.05. Descriptive statistical analyses were performed using the SPSS version 27.0 software (IBM Corp., Armonk, NY, USA) and all other analyses were conducted using R version 4.2.2 (R Foundation for Statistical Computing, Vienna, Austria).

## Results

### Participant characteristics

A total of 39 spouses were included in the present study (Table 1). The youngest participant was 26 years old, whereas the oldest was 79 years (Mdn = 61 years, IQR = 54–71 years), and the majority (*n* = 26, 67%) were women. Duration of the relationship between spouse and patient ranged from 2 to 54 years (Mdn = 34, IQR = 20–44 years). Time from the patient’s diagnosis varied from a 0 to 19 years (Mdn = 2, IQR = 1–5). Just over a third of the spouses (*n* = 15, 38%) had a university degree, the rest had attended either nine-year compulsory school or high school-level studies. Most of the participants were born in Sweden (*n* = 35, 90%). Just short of half of the participants (*n* = 17, 44%) were employed, a majority of the rest were retired (*n* = 17, 44%). Descriptively, spouses reported better physical health (Mdn = 4, IQR = 2–5) compared to psychological health and QoL (Mdn = 2, IQR = 2–2) over the past month. At the 4-week follow-up, 8 spouses declined further participation due to the worsening condition or death of the patient, 2 declined without providing a reason. The remaining participants received a follow-up questionnaire, out of which 25 completed it (Table 1).

**Table 1:** Participant characteristics at baseline and follow-up

Background variable (spouse)	Baseline (*n* = 39)	Follow-up (*n* = 25)
Age, Mdn (q1-q3) [min–max]	61 (54–71) [26–79]	63 (57–72) [26–79]
Sex, n (%)		
Female	26 (67)	13 (52)
Male	13 (33)	12 (48)
Duration of relationship with the ill patient (years), Mdn (q1-q3) [min–max]	34 (20–44) [2–54]	34 (24–44) [2–51]
Time since patient´s diagnosis (years), Mdn (q1-q3) [min–max]	2 (1–5) [0–19]	2 (1–5) [0–19]
Time since information about disease being incurable (years), Mdn (q1-q3) [min–max]	1 (1–2) [0–6]	1 (0–2) [0–6]
Education, n (%)		
University degree	15 (38)	9 (36)
High school degree or lower	24 (62)	16 (64)
Country of birth, n (%)		
Sweden	35 (90)	23 (92)
Outside of Sweden	4 (10)	2 (8)
Employment, n (%)		
Employed	17 (44)	12 (48)
Retired	17 (44)	11 (44)
Other	5 (13)	2 (8)
Physical health last month, Mdn (q1-q3) [min–max]	4 (2–5) [1–6]	3 (2–5) [1–6]
Psychological health last month, Mdn (q1-q3) [min–max]	2 (2–2) [0–5]	2 (2–2) [0–5]
Overall quality of life last month, Mdn (q1-q3) [min–max]	2 (2–4) [0–5]	2 (2–4) [0–5]
**Background variable (patient)**		
Age, Mdn (q1-q3) [min–max]	63 (55–73) [26–86]	69 (60–74) [26–78]
Sex, n (%)		
Female	12 (31)	11 (44)
Male	27 (69)	14 (56)

### Spouses’ communication with the patient about the illness and the remaining life at baseline

Over one third of the spouses talked with the patient about their thoughts and feelings concerning the illness on a daily basis (*n* = 15, 38%) (Table 2). Their answers then ranged from “less than once a week” (*n* = 8, 21%) to “three times a week” (*n* = 8, 21%). Two participants (5%) never talked about these issues with their ill partner. A majority of the spouses had talked with the patient about the illness’ impact physically and psychologically (*n* = 25, 64% and *n* = 30, 77% respectively) during the past month. Further, a majority (*n* = 25, 64%) also reported having talked about the illness being incurable. The spouses reported that they talked with the patients about the future to varying degrees. Just less than half of the participants (*n* = 18, 46%) had not discussed how to manage emotionally in the future with the patient during the past month, whereas 21 (54%) spouses reporting having had conversations with the patient about managing emotionally. Conversations about how to manage practically and financially in the future in the past month had not occurred, as reported, for a majority (*n* = 23, 59%). About one fourth (*n* = 11, 28%) reported having had these conversations “to some extent”, followed by those who answered “yes, to a great extent” (*n* = 2, 5%) and “yes, totally” (*n* = 3, 8%) (Table 2).

**Table 2:** Communication about the illness and the future at baseline

Questions	Response options	Responses at baseline
		**n (%)**
1. Do you share your thoughts and feelings with your spouse about their illness?	Never	2 (5)
	Less than once a week	8 (21)
	Once a week	6 (15)
	Three times a week	8 (21)
	Everyday	15 (38)
*Total*		*n* = *39 (100)*
2. During the past month, have you and your spouse talked about how your spouse’s illness affects him/her physically, for example with pain and nausea?	No	8 (21)
	No, but earlier	6 (15)
	Yes	25 (64)
*Total*		*n* = *39 (100)*
3. During the past month, have you and your spouse talked about how your spouse’s illness affects him/her psychologically, for example with depression and impaired thinking?	No	6 (15)
	No, but earlier	3 (8)
	Yes	30 (77)
*Total*		*n* = *39 (100)*
4. During, the past month, have you and your spouse talked about your spouse’s illness being incurable?	No	7 (18)
	No, but earlier	7 (18)
	Yes	25 (64)
*Total*		*n* = *39 (100)*
5. During the past month, have you and your spouse talked about how you will manage emotionally in the future?	No	18 (46)
	Yes, to some extent	11 (28)
	Yes, to a great extent	3 (8)
	Yes, totally	7 (18)
*Total*		*n* = *39 (100)*
6. During the past month, have you and your spouse talked about how you will manage practically and financially in the future?	No	23 (59)
	Yes, to some extent	11 (28)
	Yes, to a great extent	2 (5)
	Yes, totally	3 (8)
*Total*		*n* = *39 (100)*

### Effects of the web-based intervention

No statistically significant effects were found between the baseline and follow-up assessments in any of the questions pertaining to communication (Table 3). However, a small effect size was found in questions 1, 2. 4, 5 and 6, and a medium effect size in question 3, based on Cohen’s *r*. Although some changes in ratings were seen on individual levels, in question number 3 where the reported ratings dropped from median 3 (IQR = 2–3) to 2 (IQR = 1–3), the ratings remained stable over time.

**Table 3:** Intervention effects

	Baseline *n* = 39	Follow-up *n* = 25	*p*-value^a^	Effect size^b^
1. “Do you share your thoughts and feelings with your spouse about their illness?”				
Mdn (IQR)	4 (2–5)	4 (2–5)	0.305	0.21
2. “During the past month, have you and your spouse talked about how your spouse’s illness affects him/her psychologically, for example with depression and impaired thinking?”				
Mdn (IQR)	3 (3–3)	3 (3–3)	0.670	0.09
3. “During the past month, have you and your spouse talked about how your spouse’s illness affects him/her physically, for example with pain and nausea?”				
Mdn (IQR)	3 (2–3)	2 (1–3)	0.100	0.34
4. “During, the past month, have you and your spouse talked about your spouse’s illness being incurable?				
Mdn (IQR)	2 (2–2)	2 (2–2)	0.317	0.21
5. “During the past month, have you and your spouse talked about how you will manage emotionally in the future?”				
Mdn (IQR)	1 (1–2)	1 (1–2)	0.285	0.23
6. “During the past month, have you and your spouse talked about how you will manage practically and financially in the future?”				
Mdn (IQR)	2 (1–3)	1 (1–2)	0.773	0.16

^a^Wilcoxon signed-rank test

^b^Cohen’s *r*

## Discussion

This study set out to explore effects of a family caregiver-targeted web-based psycho-educational intervention on communication between spouses and patients with incurable illness about the illness and the remaining life in specialized home care. It is – to our best knowledge – the first in its kind to evaluate the effects of a web-based intervention on communication regarding the illness and the future with the patient from a spousal perspective. Following the web-based intervention, no statistically significant effects on the spouses’ communication with the patient about the illness and the future were found. Even if no statistically significant effect was found, a small effect size was found in all questions except for the one on whether the spouse had talked with the patient about how the illness affects them physically, where a medium effect size was found. This could be attributed to a small sample size, where not enough power was obtained to detect a statistically significant effect. A majority of the spouses had talked with the patient about different aspects of the illness and its consequences for themselves and the patient during the past month, already before having access to the web-based intervention. However, the contrary was found for communication regarding the future – where a majority of the spouses instead reported not having had these conversations at all during the past month.

In the present study, it was found that a sizeable proportion of the spouses had not ever talked about one’s own thoughts and feelings, the impact of the illness on the patient, the illness being incurable and how to manage after the patient’s death neither prior to nor following the intervention. Previous research show that conversations about the end-of-life, death, dying and future, are proven to be challenging for both family caregivers and patients with incurable illness, as found in a study on patients with cancer [34] and a recent review including the perspectives of both family caregivers and patients [19]. Similarly, the decision and will to talk or not about the patient’s illness has also been found to vary in previous research [35, 36]. Furthermore, the willingness to communicate openly about the illness and death has been shown to be higher among female spouses than male in the context of incurable cancer [37]. A majority of the spouses who enrolled in the present study were female (67%). It is possible that a study involving communication about incurable illness and the remaining life seemed more appealing to eligible female spouses than male spouses and hence, more females than males choose to enrol. Regardless, discussing the illness and the future may be challenging for both the spouse and the patient but could play an important role in ensuring that both individuals are aware of where the other person stands emotionally and practically. Further, discussions about the future may enable spouses to make important decisions regarding e.g., healthcare, legal and practical matters collaboratively with the patient.

A barrier for both family caregivers [19] and patients [34] to talk about the future is the wish to protect the other one, as well as oneself, from the distress and discomfort that such conversations might evoke. Other barriers for communication regarding the future could be avoidance of emotions generally but also avoidance of the topic of death and death-related matters specifically. This, in turn, could be attributed to e.g. fear, lack of readiness or beliefs that one needs to stay positive or that talking about death might, in fact, hasten death itself or diminish the patient’s will to fight the disease [19]. There are also factors stemming from cultural expectations and religious beliefs that could serve as barriers to communication regarding the end of life between patients and family caregivers, e.g. when the patient does not accept the diagnosis of an incurable illness, when patients or families hope for “a miracle” or for families in cultures when death is not usually talked about [38]. Given that barriers for communication between family caregivers and patients in this context are multi-faceted and multifactorial, it seems reasonable to believe that the lack of effect of the web-based intervention in the present study could possibly devolve upon barriers for communication that have not been addressed prior to, or during, the intervention. This could potentially serve as an important lesson for the continued development of this particular web-based intervention as well as future development of other psycho-educational interventions that aim to support communication about the future in similar contexts. While the development of web-based interventions aiming to support communication between family caregivers and incurably ill patients are important, it is imperative that such interventions also approach these matters sensitively. One way forward might perhaps be addressing barriers to communication directly via the intervention, encouraging family caregivers and patients to maybe engage in conversations about the barriers themselves and how to possibly overcome them. This is already done to some extent in the intervention in the present study, but greater emphasis on this could probably be beneficial. Further, interventions aimed to support communication between family caregivers and incurably ill patients also need to be culturally aware and sensitive and address the diverse needs and wishes of all involved in a variety of cultural contexts. The wide spectrum of communication barriers also underlines the need for qualitative evaluation of interventions, giving participants the opportunity to voice their experiences and potential concerns about the intervention regarding e.g. content and availability.

Prior to getting access to the website, about a third of the spouses in this study had shared their thoughts and feelings about the patient’s illness with the patient daily and a majority did so at least on a weekly basis. Even so, a large proportion of the participants also reported not having talked about how to manage emotionally and practically once the patient had passed away. Previous research shows that end-of-life communication between family members revolves heavily around providing a space for the ill patient to reflect and share their thoughts and feelings [36], which could possibly explain the lack of communication about topics revolving around the spouse’s current and future situation. A significant proportion of the spouses in the present study had not talked with the patient about neither the physical and psychological impact of the illness, nor about the illness being incurable. The same qualitative study by Jeon et al. (2023) found that within some families, family members were hesitant to address and talk about the gravity of the patient’s illness and the end of life out of concern for the patient. Challenges of end-of-life conversations for family caregivers of patients with incurable illness have also been shown in other studies [39, 40]. Sense of failure or removal of hope might hinder this kind of communication [41]. Meanwhile, recent research on communication about the illness and death between patients with incurable illness and their family members show that having had open communication about these matters was associated with reduced bereavement distress [20]. In conclusion, even though the results of the present study, alongside previous research, suggest that there are barriers to communication regarding incurable illness, end of life and death, there are clear implications for interventions that facilitate such communication while also addressing potential communication barriers.

### Methodological considerations

A strength of this study is the novelty in delivering web-based psycho-educational support to spouses of patients with incurable illness. In this study, the spouses were recruited when the patient was being cared for in specialized home care. It is to be noted that specialized home care facilities in Sweden promote a holistic approach focused on provision of support, both for the patient and the family caregivers. This might be considered a limitation, as patients and families in this context likely have access to support via their care facility that goes beyond what would be considered “standard treatment” in other care contexts. The results could have been different in another context. Another limitation is the possible skewness of the sample, where all spouses volunteered to participate in the web-based intervention, meaning they had interest in support delivered via a website. Therefore, one could speculate about whether the participants possibly represent a group with technical competence who were able to, and interested in, seeking web-based information and -support. The changes in communication that were seen on individual levels following the intervention could be attributed to a number of things as each question focuses on communication that has taken place during the past month. It is possible that communication that was potentially initiated by the website was not captured within the time window of this study. The sample size in this study is small and the study has no control group, which are limitations of the study. It is, however, the first test and evaluation of this web-based, family caregiver-targeted psycho-educational intervention and its measures, which guided the choice of design for the study. In this study, there was some attrition (*n* = 14) between baseline and the 4-week follow-up. Even though some attrition is usually expected when conducting studies within in the context of patients with incurable illness, this is a limitation of the study. The amount of missing data was very low (in total, 2 missing values for all questions), therefore no imputation was made. To summarize, larger sample sizes as well as randomized control studies to evaluate web-based psycho-educational support are warranted in the future.

## Conclusion

Even if no statistically significant effect of the web-based intervention on communication between spouses and patients was found, this study revealed that a majority of the participating spouses had talked about different aspects of the illness with the ill patient already at baseline. This might indicate that these matters are important to spouses of someone with an incurable illness. However, there was a sizeable proportion of spouses who had not communicated with the patient about the illness both prior to, and following, the intervention. An even bigger proportion of spouses reported not having ever talked to the patient about the remaining life and how to manage once the patient had passed away, neither before nor after the intervention. This might indicate that there are barriers to communication that needs to be further explored and addressed. Further, the findings of this study underline the importance of exploring, acknowledging and addressing the diverse, and perhaps varying, communication support needs of spouses of patients with incurable illnesses.

## Supplementary Information

Supplementary Material 1.

## Data Availability

The datasets generated and analysed during the current study are not publicly available due to the General Data Protection Regulations and the Swedish Ethical Review Act, but are available from the corresponding author on reasonable request
