# Supplementary material for: Communication about incurable illness and remaining life between spouses and patients with incurable illness receiving specialized home care: effects of a family caregiver-targeted web-based psycho-educational intervention
Source: BMC Palliat Care. 2024 Dec 16;23:282. doi: 10.1186/s12904-024-01614-0 (PMC11650829; doi:10.1186/s12904-024-01614-0)
Supplement: Supplementary file 1 — Supplementary Material 1. [file 12904_2024_1614_MOESM1_ESM.docx]

**Table S1. (Supplementary material).** Questions regarding communication with the patient about the illness and the future.

| **Questions** | **Response options** |
| --- | --- |
| Do you share your thoughts and feelings with your spouse about their illness? | Never |
|  | Less than once a week |
|  | Once a week |
|  | Three times a week |
|  | Everyday |
| During the past month, have you and your spouse talked about how your spouse’s illness affects him/her physically, for example with pain and nausea? | No |
|  | No, but earlier |
|  | Yes |
| During the past month, have you and your spouse talked about how your spouse’s illness affects him/her psychologically, for example with depression and impaired thinking? | No |
|  | No, but earlier |
|  | Yes |
| During, the past month, have you and your spouse talked about your spouse’s illness being incurable? | No |
|  | No, but earlier |
|  | Yes |
| During the past month, have you and your spouse talked about how you will manage emotionally in the future? | No |
|  | Yes, to some extent |
|  | Yes, to a great extent |
|  | Yes, totally |
| During the past month, have you and your spouse talked about how you will manage practically and financially in the future? | No |
|  | Yes, to some extent |
|  | Yes, to a great extent |
|  | Yes, totally |
